# Residential mobility, socioeconomic context and body mass index in a cohort of urban South African adolescents

**DOI:** 10.1016/j.healthplace.2012.09.016

**Published:** 2013-01

**Authors:** Carren Ginsburg, Paula L. Griffiths, Linda M. Richter, Shane A. Norris

**Affiliations:** **a** MRC/Wits Developmental Pathways for Health Research Unit, Department of Paediatrics, Faculty of Health Sciences, University of the Witwatersrand, Johannesburg, South Africa; b School of Sport, Exercise and Health Sciences, Loughborough University, LE, UK; c Human Sciences Research Council, South Africa

**Keywords:** BMI, Residential mobility, Transitioning socioeconomic status, Urban adolescents, South Africa

## Abstract

Adolescents who are changing residence, as well as their social and economic circumstances may experience lifestyle changes that have an effect on body composition outcomes such as undernutrition, overweight or obesity. This paper uses data from Birth to Twenty, a birth cohort of South African urban children, to determine the relationship between residential mobility and body mass index (BMI) amongst Black adolescents aged 15 (*n*=1613), and to examine the role of changes in household socioeconomic status (SES). The prevalence of overweight and obesity in the sample was 25% in females and 8% in males. Amongst the females, a strong positive association between residential mobility and BMI was observed for those who also experienced an increase in household SES between birth and 15 years (*β*=0.42, SE=0.13), while no effect was identified for males. The study shows the potential for environmental change and increased resources to influence the risk for obesity. It also highlights the value in considering the range of social environmental factors and changes across the early life course that might play a part in evolving nutritional patterns in urban transitioning environments.

## Highlights

► Previous studies have not considered associations between mobility, SES & obesity from birth to age 15. ► Risks for obesity in this urban transitioning environment differ between groups of adolescents. ► Findings show that residential mobility together with rising SES increase the risk for obesity. ► Cross sectional socio-demographic measures miss the complexity of changing environments.

## 1 Introduction

The prevalence of overweight and obesity is increasing in low- and middle-income countries (LMICs). Urbanisation and economic development have resulted in a nutrition transition characterised by a shift to a higher caloric diet and/or a reduction of physical activity (Popkin, 2003). While some populations continue to experience undernutrition, escalating levels of obesity have been observed amongst both higher, and increasingly lower, socioeconomic groups in countries across the globe (Monteiro et al., 2004, Popkin, 1999). In many LMICs this rising prevalence of overweight and obesity is experienced concurrently with persistent high levels of undernutrition, meaning that such societies are double burdened (Varela-Silva et al., 2012). Within the African region, overweight and obesity are associated with a raised incidence of non-communicable diseases (NCDs), especially cardiovascular disease (Popkin and Doak, 1998). It is anticipated that by the year 2020, deaths resulting from NCDs will increase by over 20% on the African continent (World Health Organization, 2011). Thus there is a strong incentive to understand the environmental and lifestyle factors and processes that may be associated with body composition changes and in particular, risk of developing obesity within these settings.

In LMICs, the process of urbanisation has been highlighted as a key contributor to the nutrition transition (Vorster et al., 1999). This is because movement to urban areas is associated with changes in dietary patterns and lifestyles which may lead to overweight and obesity (Popkin and Gordon-Larsen, 2004). With the United Nations predicting a significant increase in the rates of urbanisation and internal migration within the African continent over the coming decades, dynamics associated with population mobility are important in order to facilitate a broader understanding of the nutrition transition (United Nations, 2010). Population movement may take a variety of forms, occur over a range of distances, and with varying degrees of permanence (Boyle et al., 1998). According to the theory of migration selection, movement of individuals may be prompted by the search for opportunities and improved socioeconomic conditions (pull factors), or driven by adversities or difficulties experienced at locations of origin (push factors) (Lee, 1966). Whether pull or push, relocation results in an altered set of environmental and socioeconomic conditions which may have either positive or negative consequences for individuals’ health and well-being (Brockerhoff, 1990, Garenne, 2006).

It is well established that nutrition interventions need to focus on the complex interactions between ecological factors involving the individual within their interpersonal, community and societal context, because all of these levels are important in influencing body composition outcomes (McLeroy et al., 1988). Movement of an individual from one context to another, even within a small geographical area, would result in changes in community, facilities (e.g. parks and open spaces), food purchasing opportunities and access, and interpersonal relationships (Lopez and Hynes, 2006). Thus it follows that relocation may influence body composition and risk for poor nutrition outcomes such as overweight and obesity due to shifts in diet, health practices, or behaviour brought about by such changes in environment (Zezza et al., 2011). Establishing causality is complex, and the relationship between mobility and body composition may be mediated by a number of factors. In particular, the effects of changes resulting from movement are likely to be felt differently among different socioeconomic groups. These groups have different levels of access to individual and household resources that influence the way that the environment and changes in environment are experienced. For example, in a study of international migrants it was found that movement alone did not results in obesity unless it was also associated with improved socioeconomic conditions (Renzaho, 2004). This is because such conditions are required to promote the consumption of energy dense diets and more sedentary lifestyles. While the relationship between SES and body composition has been well documented (McLaren, 2007, Sobal and Stunkard, 1989), previous studies have not expressly considered the relationships concurrently between changes in residence, SES and body composition in the African context.

South Africa provides an appropriate setting in which to investigate patterns of mobility and corresponding health outcomes within the context of individuals’ socioeconomic environments. An urban transition is underway in post-Apartheid South Africa, which is characterised by unique patterns of population mobility to and within urban areas (Collinson et al., 2007). High levels of urbanisation have been documented particularly amongst Black South Africans, with temporary and circular mobility also common (Kok and Collinson, 2006). Such internal mobility is strongly linked to socioeconomic circumstances with movement associated with both higher and lower levels of resources (Kok et al., 2003). Relocation may occur both in the case of adults and amongst children or adolescents who might move independently of a parent or primary caregiver for a variety of reasons linked to children’s own circumstances (e.g. to gain access to school), or for reasons prompted by a connected adult (Kok and Collinson, 2006, Van der Waal, 1996). Children and adolescents are particularly vulnerable to changes of residence and numerous international studies have linked residential mobility among children to a range of negative health and social consequences (Jelleyman and Spencer, 2008). However, no South African research has focused on the role of such changes in environment on risk factors for poor health outcomes such as overweight or underweight. With studies identifying an increased prevalence of overweight among females during late childhood and adolescence (Kimani-Murage et al., 2011, Reddy et al., 2009), further empirical research is needed to disentangle the risk factors and potential positive impacts of internal mobility on health among South African children and adolescents. Given the differing levels of resources and inequality in incomes which persist in the South African context (May, 2000) and the transitory nature of SES within this setting (May and Woolard, 2007), the possible ways in which SES may alter these relationships is likely to be significant.

Against the background outlined, the aim of this paper is to determine the association between residential mobility and BMI, and to establish whether the association is moderated by the effect of changing household SES in a cohort of South African urban adolescents aged 15.

## 2 Methods

### 2.1 Study sample

The paper uses data from Birth to Twenty (Bt20), a longitudinal birth cohort study of urban South African children. Bt20 commenced at the onset of South Africa’s transition to democracy in 1990 with the enrolment of a cohort of singleton children born during a seven-week period in the Greater Johannesburg area in the Gauteng Province. Of the 5449 births that took place during the period, a sample of 3273 children identified from the total as permanently resident in the area were recruited into the longitudinal birth cohort (Richter et al., 2004). The main aim of Bt20 was to study children’s physical and social development in an environment of rapid social change (Richter et al., 2007). The Gauteng Province was selected because it is South Africa’s most densely populated urban area, home to approximately 10.5 million residents (Statistics South Africa, 2009). This region provides the ideal transitioning environment to study child health, well-being and household environments.

At enrolment, the Bt20 cohort had similar proportions of males (48.6%) and females (51.4%). The cohort were predominantly Black (78.5%); with White, Coloured, and Asian children comprising 6.3%, 11.7% and 3.5% of the cohort respectively, which was roughly representative of the population proportions at the time. At birth, 10.8% of children were considered underweight (<2.5 kgs). Characteristics of the biological mothers of the cohort members show that the majority were single (56.5%), and most had not completed secondary school (58.4%). Data collection activities among the cohort have taken place at intervals of either one or two years, beginning with questionnaires administered antenatally to pregnant women. Previously reported retention statistics for the cohort indicate that the Bt20 study had maintained contact with 70% of the cohort by the start of the 16th year (Norris et al., 2007). Reasons why participants were lost to follow-up include caregiver or child mortality, study fatigue or movement out of the study area.

During the study’s 15th year, a residential mobility questionnaire was administered to all participants in contact with Bt20. The questionnaire aimed to verify all historical address records as correctly reflecting the children’s primary places of residence over the 15 year period, and to complete any missing or partial address information. The questionnaire data enabled the construction of a residential history for each child from birth to 15 years from which movements could be identified (see Ginsburg et al., 2009 for a more detailed description of the study of residential mobility within the cohort). During the course of this same year, anthropometric assessments of the cohort were also conducted. The current analytical sample comprises those cohort participants who completed the residential mobility questionnaire at age 15, and had growth (weight and height) measurements taken at age 15. The sample was restricted to Black participants (*n*=1613) as this is the population most affected by the nutrition transition, and smaller numbers of participants from the other ethnic groups meant that these relationships could not be fully investigated within these sub-groups. The available samples for White, Coloured and Asian participants with complete data numbered 46, 200 and 24, respectively.

### 2.2 Variables

For the purposes of analyses, BMI Z-scores were derived using the WHO reference (World Health Organization, 2009). Raw BMI measures were also categorised using the age and sex appropriate classifications proposed by Cole et al., 2000, Cole et al., 2007 for underweight, overweight and obesity. The models included a measure describing the onset of puberty which was defined as age of menarche for females and age of transition from Tanner 1 to Tanner 2 for males (Tanner, 1962). Residential mobility was analysed using a binary variable contrasting those cohort participants who had moved one or more times over their first 15 years, with those who had never experienced a residential move over the same time frame. Mobility was also investigated using a variable with three categories representing different frequencies of movement over the time period (those who had never moved, those who had moved once and those who had moved two or more times). The magnitude and significance of the results were found to be consistent with both mobility measures and in the interests of presenting a more parsimonious set of models, the results using the binary measure of mobility only have been reported here. Household SES was assessed on the basis of access to a set of ten assets and services (house type, home ownership, access to indoor water, access to a flush toilet, access to electricity and ownership of a television, motor vehicle, refrigerator, washing machine and telephone). Socioeconomic data were available for two time points, one at the birth of the Bt20 child and another when the participant was aged between 12 and 13 years. For each time point, an index was calculated using a probit factor model and estimated factor loadings for each item were used to represent a “wealth” measure (see Ginsburg et al., 2011 for further details of this measure). On the basis of these scores, participants were grouped into tertiles representing low, medium and high SES. Change in SES between birth and age 12/13 was determined by comparing the resulting categorical tertile variables across the two time points and assessing whether a participant’s socioeconomic position had decreased, increased or remained the same. An interaction between residential mobility and initial as well as change in SES was developed to allow for any moderating effects of these variables to be included in analyses. The analyses also included demographic data describing characteristics of cohort members’ biological mothers or primary caregivers. These included a variable representing the change in primary caregivers’ marital status between the time of the birth of the cohort child and the time that the cohort participant turned 13 years old. A categorical measure of maternal education and maternal age at the time of the birth of the cohort participant were also controlled for in the models. These variables were included in order that broader elements of the participant’s social contexts were considered in the analyses.

### 2.3 Statistical analysis

Descriptive statistics were used to define the sample characteristics. Multiple regression analysis was employed to investigate the association between residential mobility and BMI Z-scores, and to determine if the association differed between socioeconomic groups. The first regression analysis considered the unadjusted effect of mobility on BMI (Model 1). This was followed by a model which investigated the unadjusted effect of changing SES on BMI, controlling for initial levels of SES (Model 2). The third unadjusted regression model explored the combined effects of residential mobility and SES on BMI using a variable representing residential mobility interacted with change in SES, and controlling for SES at birth (Model 3). In order to investigate whether mobility effects may be explained by changing socioeconomic circumstances, a first adjusted model regressed both residential mobility and changing SES on BMI without controlling for other possible confounders (Model 4). Further models were than adjusted to control for other potentially confounding factors described in the variables section. The first of these adjusted models aimed to test the effects of SES at birth and changing SES on BMI (Model 5), while the second explored the interaction between mobility and SES, adjusting for early childhood SES (Model 6). Models were stratified by sex because of differences in BMI driven by puberty at this age. In order to assess whether there were any significant differences between the analysis sample and the original Black participants in the cohort, a comparison between the analytical sample members and those participants who were excluded from the analytical sample was conducted using t-tests for continuous variables and the multi-dimensional Chi-square tests for categorical variables. All analyses were undertaken using IBM SPSS 19.0 and alpha level error was set at 0.05.

## 3 Results

The comparison of the analytical sample and those Black participants who were excluded because of missing data revealed that the two samples were equivalent in relation to sex and birth weight. The analytical sample members were significantly more likely to have had biological mothers who were single at the time of their birth and had completed a Grade 11–12 education, as compared with a higher representation of married mothers and mothers with below Grade 10 education in the sample of excluded participants. The Black participants excluded from the analytical sample had a significantly lower mean SES index as compared with those in the analysis sample (see Table 1).

**Table 1:** Characteristics of members of the analytical sample and cohort members excluded from the analytical sample (limited to Black participants).

		**Analytical sample** (%) *n*=1613	**Bt20 Black participants excluded from analysis** (%) *n* =955
**Child sex**	Male	773 (47.9)	473 (49.5)
*χ*_(1)_^2^=0.619, NS, *n* =2568	Female	840 (52.1)	482 (50.5)

**Birthweight**	<2.5 kg	201 (12.5)	95 (9.9)
*χ*_(1)_^2^=3.716, NS, *n* =2568	≥2.5 kg	1412 (87.5)	860 (90.1)

**Parity**	1	625 (38.7)	306 (32.0)
	2	478 (29.6)	291 (30.5)
	3	269 (16.7)	188 (19.7)
*χ*_(3)_^2^=13.698, *p* <0.01, *n* =2568	4+	241 (14.9)	170 (17.8)

**Maternal age at delivery**	≤18	207 (12.8)	90 (9.4)
	19–34	1237 (76.7)	774 (81.0)
	35+	169 (10.5)	90 (9.4)
*χ*_(3)_^2^=9.831, *p* <0.05, *n* =2568	Missing	0 (0.0)	1 (0.1)

**Maternal education at delivery**	Grade 10 or less	202 (12.5)	219 (22.9)
	Grade 11–12	1200 (74.4)	556 (58.2)
	Post-school training	114 (7.1)	69 (7.2)
*χ*_(3)_^2^=85.918, *p* <0.001, *n* =2568	Missing	97 (6.0)	111 (11.6)

**Maternal marital status at delivery**	Married/living with partner	523 (32.4)	405 (42.4)
	Single/widowed/divorced/separated	1085 (67.3)	546 (57.2)
*χ*_(2)_^2^=26.372, *p* <0.001, *n* =2568	Missing	5 (0.3)	4 (0.4)

**Household socioeconomic index at birth**	Mean (SD)	−0.31 (0.72)	−0.60 (0.82)
*t*_(2513)_=9.339, *p* <0.001, *n* =2515			

The anthropometric and demographic profile of males and females in the sample is presented in Table 2. The mean BMI Z-scores for males in the sample was −0.47 (SD=1.16, *n*=773), while females in the sample had mean BMI Z-scores of 0.32 (SD=1.19, *n*=840). The majority of males in the sample had BMIs within the normal range (72%), however a fifth of the sample were classified as underweight. A large proportion of females in the sample were classified as overweight (18%) or obese (8%). More than half of the sample had experienced at least one residential move within their first 15 years; the others, 47% male and 43% female, had never moved home. The maximum number of moves over the time period was 6 (*n*=2), however, the majority of movers in the sample (57%, *n*=500) had changed residence only once and only 13% (*n*=117) of the mobile group had relocated more than twice. Approximately one quarter of the sample had improved household SES between birth and the age of 15, while over a quarter of the sample had experienced a drop in relative household SES over this time frame.

**Table 2:** Descriptive statistics relating to Black Bt20 cohort participants at age 15 years.

		**Male** (%) *n*=773	**Female** (%) *n*=840
**Anthropometric measures**			
BMI Z-scores		−0.47 (1.16)	0.32 (1.19)
BMI^a^	Underweight	157 (20.3)	81 (9.6)
	Normal	555 (71.8)	549 (65.4)
	Overweight	42 (5.4)	147 (17.5)
	Obese	19 (2.5)	63 (7.5)
Birthweight	<2.5 kg	85 (11.0)	116 (13.8)
	≥2.5 kg	688 (89.0)	724 (86.2)
Parity	1	287 (37.1)	338 (40.2)
	2	231 (29.9)	247 (29.4)
	3	128 (16.6)	141 (16.8)
	4+	127 (16.4)	114 (13.6)
Age of menarche %	≤11 years	∼	138 (16.4)
	12–13 years	∼	495 (58.9)
	14+ years	∼	202 (24.0)
	Missing	∼	5 (0.6)
Age of initiation of puberty (movement from Tanner stage 1 to Tanner stage 2)	<12 years	261 (33.8)	∼
	12–13 years	358 (46.3)	∼
	14+ years	149 (19.3)	∼
	Missing	5 (0.6)	∼
**Mobility and SES**			
Total residential moves	Never moved by age 15	365 (47.2)	364 (43.3)
	≥1 move by age 15	408 (52.8)	476 (56.7)
SES change	Decreased	230 (29.8)	238 (28.3)
	No change	348 (45.0)	374 (44.5)
	Increased	195 (25.2)	228 (27.1)
Mobility and SES interaction	No move, decrease in SES	121 (15.7)	116 (13.8)
	No move no change in SES	183 (23.7)	184 (21.9)
	No move, increase in SES	61 (7.9)	64 (7.6)
	Move, decrease in SES	109 (14.1)	122 (14.5)
	Move, no change in SES	165 (21.3)	190 (22.6)
	Move, increase in SES	134 (17.3)	164 (19.5)
**Maternal characteristics**			
Maternal age at delivery	≤18	98 (12.7)	109 (13.0)
	19–34	593 (76.7)	644 (76.7)
	35+	82 (10.6)	87 (10.4)
Maternal education at delivery	Grade 10 or less	96 (12.4)	106 (12.6)
	Grade 11–12	571 (73.9)	629 (74.9)
	Post-school training	57 (7.4)	57 (6.8)
	Missing	49 (6.3)	48 (5.7)
Maternal marital status at delivery	Married/living with partner	267 (34.5)	256 (30.5)
	Single/widowed/divorced/separated	504 (65.2)	581 (69.2)
	Missing	2 (0.3)	3 (0.4)
Change in primary caregiver marital status	Married stayed married	141 (18.2)	123 (14.6)
	Married to single	75 (9.7)	88 (10.5)
	Single to married	154 (19.9)	182 (21.7)
	Single stayed single	242 (31.3)	291 (34.6)
	Missing	161 (20.8)	156 (18.6)

a For males aged 15.5 years, underweight was defined as those with BMI ≤17.26, normal: BMI >17.26 and <23.6, overweight: BMI ≥23.6 and <28.6, obese: BMI ≥28.6 For females aged 15.5 years underweight was defined as those with BMI ≤17.69, normal: BMI >17.69 and <24.17, overweight: BMI ≥24.17 and <29.29, obese: BMI ≥29.29 (see Cole et al., 2000, Cole et al., 2007).

Table 3 presents the results of three unadjusted multiple regression analyses of BMI Z-scores, with residential mobility and SES. For males, no effect of mobility, changing SES or an interaction of these factors was observed. Amongst the females in the sample, Models 1 and 2 revealed that both residential mobility and a shift to higher SES were independently associated with BMI in a positive direction. Combining the effects of these variables in Model 3 resulted in a significant positive association between residential mobility and BMI for those females in the sample who also experienced an increase in household SES between birth and 15 years. For this group, BMI Z-scores were 0.45 units higher on average than their counterparts who had not changed SES and not moved. For males, an unadjusted model of the early life household SES regressed on BMI Z-scores revealed a significant positive association between the dependent variable and those in the highest SES tertile at birth (*β*=0.25, SE=0.11, *p* <0.05), while no significant association was present in the case of females (*β*=0.06, SE=0.11, NS, *p*=0.61, models not shown).

**Table 3:** Multiple linear regression analyses: BMI Z-scores - unadjusted models.

	**Model 1**				**Model 2**				**Model 3**			
	Males		Females		Males		Females		Males		Females	
	*β*	SE	*β*	SE	*β*	SE	*β*	SE	*β*	SE	*β*	SE
Total residential moves					∼	∼	∼	∼	∼	∼	∼	∼
(Never moved by age 15)												
≥1 move by age 15	0.047	0.083	0.166^c^	0.083								
SES at birth	∼	∼	∼	∼								
(Lowest tertile)												
Middle tertile					0.154	0.108	0.142	0.105	0.155	0.107	0.146	0.105
Highest tertile					0.233	0.123	0.189	0.120	0.229	0.123	0.195	0.120
SES change	∼	∼	∼	∼					∼	∼	∼	∼
(No change)												
Decreased					−0.083	0.108	−0.070	0.107				
Increased					−0.117	0.108	0.243^c^	0.106				
Mobility*SES	∼	∼	∼	∼	∼	∼	∼	∼				
(No move, no change in SES)												
No move, decrease in SES									−0.233	0.142	0.092	0.147
No move, increase in SES									−0.114	0.174	0.025	0.175
Move, decrease in SES									0.062	0.146	−0.057	0.144
Move, no change in SES									−0.019	0.124	0.171	0.122
Move, increase in SES									−0.134	0.135	0.450^b^	0.132
Constant	−0.496^a^	0.061	0.221^a^	0.062	−0.538^a^	0.085	0.160	0.086	−0.528^a^	0.104	0.070	0.106
Adjusted R^2^	−0.001		0.004		0.003		0.005		0.004		0.012	

a *p*<0.001.

b *p*<0.01.

c *p*<0.05.

The results of the model exploring the independent effects of mobility and changing SES, together with the two models adjusted for confounders are presented in Table 4. Model 4 reveals that prior to controlling for any other explanatory variables, an increase in SES was significantly positively associated with BMI for females (*β*=0.21, SE=0.11). For males, there were no significant relationships between BMI Z-scores and residential mobility or household SES in any of the models. However, males who were born of higher parity (*β*=−0.27, SE=0.11 for those with parity 2 and *β*=−0.29, SE=0.14 for those with parity 3), with birthweights below 2.5 kgs (*β*=−0.34, SE=0.13) and whose mothers had not completed post-school education (*β*=−0.41, SE=0.20 for mothers with Grade 10 or less schooling and *β*=−0.43, SE=0.16 for mothers with Grade 11–12 schooling), were significantly more likely to have lower BMI Z-scores (see Model 6). In contrast to the models for males, the female adjusted regression models revealed that none of the maternal factors were significant in explaining BMI. The significant positive relationship between BMI and improved SES (*β*=0.23, SE=0.11, Model 5), and the interaction between residential mobility and improved household SES remained in the adjusted models (*β*=0.42, SE=0.13, Model 6). The significant findings for females were also checked in a logistic regression model of the clinical cut off for overweight and obesity and the same significant pattern of association persisted (95% CI for females who had moved residence and increased SES (1.208, 3.711), OR 2.117 compared to those who had not moved and not changed SES).

**Table 4:** Multiple linear regression analyses: BMI Z-scores - adjusted models.

	**Model 4**				**Model 5**				**Model 6**			
	Males		Females		Males		Females		Males		Females	
	*β*	SE	*β*	SE	*β*	SE	*β*	SE	*β*	SE	*β*	SE
Total residential moves									∼	∼	∼	∼
(Never moved by age 15)												
≥1 move by age 15	0.077	0.085	0.134	0.084	0.078	0.087	0.085	0.084				

SES at birth												
(Lowest tertile)												
Middle tertile	0.156	0.108	0.146	0.105	0.111	0.110	0.082	0.105	0.106	0.110	0.082	0.104
Highest tertile	0.234	0.123	0.187	0.120	0.159	0.127	0.042	0.124	0.147	0.127	0.049	0.124

SES change									∼	∼	∼	∼
(No change)												
Decreased	−0.084	0.108	−0.072	0.107	−0.066	0.109	0.047	0.107				
Increased	−0.134	0.110	0.213^c^	0.107	−0.158	0.110	0.232^c^	0.105				

Mobility*SES	∼	∼	∼	∼	∼	∼	∼	∼				
(No move, no change in SES)												
No move, decrease in SES									−0.255	0.143	0.200	0.144
No move, increase in SES									−0.167	0.175	−0.042	0.172
Move, decrease in SES									0.090	0.147	−0.015	0.143
Move, no change in SES									−0.056	0.126	0.081	0.122
Move, increase in SES									−0.173	0.138	0.422^b^	0.132

Birthweight	∼	∼	∼	∼								
(≥2.5 kg)												
<2.5 kg					−0.331^c^	0.133	−0.199	0.116	−0.341^c^	0.133	−0.202	0.116

Parity	∼	∼	∼	∼								
(1)												
2					−0.256^c^	0.111	0.240^c^	0.107	−0.268^c^	0.112	0.233^c^	0.107
3					−0.267	0.136	0.128	0.133	−0.285^c^	0.136	0.139	0.132
4+					−0.250	0.160	0.423^b^	0.158	−0.266	0.160	0.426^b^	0.158

Age of menarche	∼	∼	∼	∼	∼	∼			∼	∼		
(12–13 years)												
≤11 years							0.294^b^	0.113			0.290^c^	0.112
14+ years							−0.632^a^	0.098			−0.646^a^	0.097
Missing							−0.518	0.525			−0.507	0.523

Onset of puberty	∼	∼	∼	∼			∼	∼			∼	∼
(12–13 years)												
<12 years					0.174	0.096			0.180	0.096		
14+ years					−0.031	0.119			−0.029	0.119		

Maternal age at delivery	∼	∼	∼	∼								
(≤18)												
19–34					0.143	0.141	−0.150	0.135	0.156	0.141	−0.151	0.134
35+					−0.015	0.211	−0.111	0.200	0.002	0.211	−0.117	0.200

Maternal education at delivery	∼	∼	∼	∼								
(Post-school training)												
Grade 10 or less					−0.386	0.199	−0.142	0.204	−0.406^c^	0.199	−0.142	0.203
Grade 11–12					−0.418^c^	0.162	0.031	0.165	−0.432^b^	0.162	0.046	0.164
Missing					−0.326	0.227	−0.156	0.229	−0.307	0.227	−0.168	0.228

Change in primary caregiver marital status	∼	∼	∼	∼								
(Married stayed married)												
Married to single					−0.291	0.166	−0.083	0.162	−0.281	0.166	−0.099	0.161
Single to married					−0.139	0.144	0.070	0.143	−0.139	0.144	0.073	0.142
Single stayed single					−0.187	0.133	0.017	0.135	−0.177	0.133	0.006	0.135
Missing					−0.154	0.144	0.091	0.144	−0.152	0.144	0.095	0.144
Constant	−0.575^a^	0.095	0.091	0.096	0.024	0.252	0.229	0.253	0.096	0.255	0.229	0.256
Adjusted R^2^	0.003		0.007		0.023		0.074		0.026		0.082	

a *p*< 0.001.

b *p*< 0.01.

c *p*< 0.05.

## 4 Discussion

This is the first South African study to explore the associations between residential mobility, SES and BMI. The results of this study reveal that both residential mobility and changing SES independently confer risk for increased BMI amongst females in this setting, and SES has both a mediating and a moderating effect on the relationship between residential mobility and BMI. The study found, for the first time in an urban African context, that the combined experience of increasing SES along with mobility creates the highest risk for raised BMI for female adolescents with high prevalence of overweight. The study illustrates that for males, underweight is a more dominant problem suggesting that urban transitioning environments are complex and not all groups share the same risks.

The nutrition transition describes a shift to diets and lifestyles that are conducive to overweight and are often associated with urban living. Indeed findings from the South African Demographic and Health Survey (DHS) and the National Food Consumption Survey indicate that the prevalence of obesity is higher in urban areas as compared with rural areas of the country, particularly amongst Black females (Kruger et al., 2005, Puoane et al., 2002). Nationally, the prevalence of overweight in South African Black males is below that of females with the South African DHS reporting a prevalence of obesity of 7.7% in Black adult males and 30.5% in Black adult females (Puoane et al., 2002). The high levels of overweight in females have been explained by factors such as unhealthy eating and sedentary activity; environmental conditions which are associated with urban living (Feeley et al., 2012, Kruger et al., 2005, Puoane et al., 2002). For males it has been suggested that lifestyle factors such as more active employment or higher levels of physical activity are protective against obesity (Department of Sport and Recreation, 2005, Kruger et al., 2006).

The findings of the present study corroborate these trends by demonstrating a marked variation in the patterns of BMI by sex. For adolescent males within the cohort, the greater prevalence of underweight is consistent with data from other South African studies of youth (Reddy et al., 2010). In the present analysis, underweight appears to be driven more strongly by early life factors such as SES at birth and maternal environment (although it is possible that the risk for overweight in males may emerge at a later developmental stage). For females, the rates of overweight and obesity are high, again mirroring South African trends in adolescents (Kimani-Murage et al., 2011, Reddy et al., 2010). Within the cohort, accelerated weight gain has been observed following the onset of puberty (the prevalence of overweight among Black females at age 9 was 10%) (Griffiths et al., 2008). Given the observed association with changes in household resources and residential location, it follows that altered environmental contexts may be particularly critical for females during adolescence.

This study revealed a positive association between BMI and household SES, which is indicative of the process of transition that is underway in this urban population. While the lowest socioeconomic groups appear to be less susceptible to overweight, those who are shifting from lower to higher relative SES are at higher risk compared with all other groups. This positive relationship between SES and overweight has been observed in a nationally representative study of overweight amongst South African urban youth, as well as other South African studies (Kimani-Murage et al., 2011, Reddy et al., 2012, Steyn et al., 2005), although the present study is the first of these to have established a relationship between increased BMI and increasing SES over time. The literature highlights a trend towards obesity among the lower SES groups of many LMICs (Monteiro et al., 2004, Popkin and Gordon-Larsen, 2004, Prentice, 2006). Such a trend has not yet been observed in this urban South African cohort with the highest SES groups still having the highest risk of obesity. The lack of a transition towards the lowest SES groups likely reflects the fact that this sample represents a lower socioeconomic sector of the South African urban population, where the relatively more wealthy within this lower SES group are experiencing the effects of transition first.

This study has also identified a complex relationship between residential mobility, SES and BMI. Results show that improving SES in the first 15 years of life mediates the significant effect of a residential move on BMI for females suggesting that the mobility effect on BMI is explained by the improving SES of the movers. However, results also show that there is a moderating effect of SES on the association between movement and BMI, such that relocation is only significantly associated with increased BMI in females if it is also coupled with an improving SES profile in the first 15 years of life. Relocation has been shown to heighten the risk of overweight because of the potential for new environments to allow for greater access to energy-dense foods coupled with lower physical activity (Kruger et al., 2005, Popkin and Gordon-Larsen, 2004). It thus follows that adolescents who are changing residence as well as SES are likely to be those who are dealing with the most rapid exposure to new lifestyles that place them at greatest risk of obesity. Within the Bt20 study population, residential mobility is strongly linked to socioeconomic factors with movement found to be employed as a strategy to either improve children and families’ living circumstances, or to survive in challenging or prohibitive conditions (Ginsburg et al., 2009, Ginsburg et al., 2011). The present study highlights that in the case of mobility prompted by improved circumstances, there is the potential for adverse consequences. Internal and intra-urban mobility in South Africa is increasing and is particularly prevalent during the young adult years where relocation is often driven by the search for employment, educational opportunities, relationship changes or housing access (Cross, 2006, Kok et al., 2003). It may be anticipated that the transition to adulthood would present a critical period in which environmental changes and associated effects on health outcomes might be observed.

The results of this research yield insight into the role of transitioning environments on the risk for overweight and obesity, however a number of study limitations and opportunities for future research should also be highlighted. The relationship between mobility and health is complex and may be influenced by the form the movement takes, the circumstances driving and resulting from the movement, as well as a range of confounders at the environmental and individual levels (Collinson et al., 2006, Garenne, 2006). In order to develop a more thorough understanding of the pathways through which mobility may influence health, access to a wide variety of data would be necessary. Although the current study assisted in exploring some of these links, a number of confounders and explanatory factors could not be examined because of a lack of suitable data. These include descriptors at the neighbourhood or community level, which would provide insight into the specific environmental changes resulting from movement. Reasons or circumstances prompting movement would also be of value in informing the context in which moves took place. In the present study, residential mobility refers to all moves between birth and the age of 15 and does not consider smaller time intervals. This analysis could be extended to explore the effects of the timing of a residential move on changes in body composition using longitudinal modelling techniques. Such an application would provide insight into whether mobile individuals experience greater than expected changes in body composition. The analysis of mobility may further be strengthened by incorporating a distance measure of movement, and using graphical mapping tools to further investigate the relationships outlined here. Also, given that levels of physical activity and dietary composition are key determinants of obesity, an investigation of the association between mobility, SES, and these lifestyle behaviours would add important insight into the factors on the causal pathway between social environment and obesity.

A final limitation of the present study relates to those cohort members who were excluded from the analytical sample due to attrition or missing data. Attrition is a concern in a cohort study of mobility as drop-out is often associated with the outcome of interest. Indeed, many of the untraceable Bt20 participants were lost to follow-up because of frequent circular mobility or movement out of the study area, and amongst this group was a higher representation of those from the lowest resourced households (Ginsburg et al., 2009). By virtue of their different characteristics, the relationships observed in this paper may differ for the group of excluded participants in contrast with those who continued participation in Bt20. It is conceivable that amongst this vulnerable group, higher levels of mobility coupled with lower relative socioeconomic conditions could present a greater risk of undernutrition due to food insecurity. Targeted research aimed at exploring body composition outcomes amongst the more poverty stricken or disadvantaged urban youth would be of value.

In conclusion, this paper is unique in being able to show the potential for environmental change and increased resources to influence the risk for obesity in transitioning societies, in particular for females. The relationship between mobility, changing SES and BMI is however complex. Understanding the health consequences associated with the nutrition transition has been identified as a research priority in South Africa and other LMICs where implications in relation to health policy may be significant (Popkin, 2002, Reddy et al., 2012). This research contributes to the development of such understanding and highlights the value in considering the range of social environmental factors and changes in these factors in the early life course that might play a part in evolving nutritional patterns in urban transitioning societies. Mobility and associated social dynamics require exploration over time and longitudinal study designs such as cohort and panel studies provide an important source of data for undertaking such investigations. Focusing research on cross-sectional analyses in transitioning environments misses important changes in the social environment over the short term, as well as the transitioning nutrition and health environments. Following from this study, it is critical that new research is undertaken to understand the mechanisms through which mobility, and changing social and environmental factors influence behaviours that place individuals and households at risk of poor nutrition outcomes, so as to better target interventions.
